# Vascular architecture of the monocot *Cyperus involucratus* Rottb. (Cyperaceae)

**DOI:** 10.1186/s40064-015-1641-z

**Published:** 2016-01-04

**Authors:** Robert W. Korn

**Affiliations:** Department of Biology, Bellarmine University, 2001 Newburg Rd., Louisville, 40205 USA

**Keywords:** Atactostele, Ordered patterns, Parallel veins

## Abstract

The arrangement of vascular bundles in the stems of monocots has been described repeatedly as “scattered.” But to the trained eye it is clearly ordered as verified by the use of the R index of Clark and Evans. The arrangement of bundles in leaves and sclerenchyma bundles in stems are also ordered. An equation was developed for the probability distribution frequencies (pdf) for leaf intervein distances which curiously also fits for cell size in proliferating tissues. Another equation was developed for the pdf for intervein distances in stems which can also be applied to epidermal deriviatives such as stomata and trichomes.

## Background

Description of plant structure, namely, morphology and anatomy, can be carried out with different degrees of precision. The simplest method is that of qualitative geometry, next is that of data collection and then mathematically by an equation, the last of which should generate data comparable to that from actual tissue. Finally a computer graphics program is developed that depicts the geometric description. For example, cell proliferation can be detailed by these four levels (Korn 2001). Geometrically cell arrays are cellular networks composed of space filling polygons. Next, data can be collected as to size of cells either in one, two or three dimensions and given as a probability density function (pdf). Then an equation can be formulated that generates a pdf of cell sizes similar to that from real data. From the geometric description data can also be collected on shape, namely, number and length of walls. Together cell size and shape can be joined into a computer graphics program. Later cell specialization can be added such as stomatal and trichome formation (Korn 1993).

The venation pattern in monocots is an interesting example that has hardly passed beyond the simplistic geometric description. Numerous references to leaf venation note the geometric feature of parallel venation in monocots as opposed to the network arrangement of veins in dicot leaves (Nelson and Dengler 1997) with only a suggestion of possible molecular models since little tissue data is available on which to test specific models. Geometry of the monocot stem vasculature is equally vague. George Brebner (1902) coined the term atactostele (Greek *atact*—without order) for vein arrangement seen in transverse view which has been described later as “scattered” by Berg (1997), Purves et al.(2003) and countless others implying no pattern is present. Mauseth (1988) noted the only venation pattern in stems seen in 3D is parallel venation. Casual observation of the transverse view of a monocot stem (Kumazawa 1961, Fig. 3) indicates to the informed eye veins are not scattered but appear to be ordered as no case is seen of two parallel veins deployed close to each other, such as by less than their diameter. *Cyperus involucratus* Rottb. was selected as the monocot of study for two reasons. First, it has the typical parallel venation pattern in stems and leaves and, second, the long internode, or scape, of the culm is free of any complications of leaf traces.

## Results and discussion

**Fig. 1:**
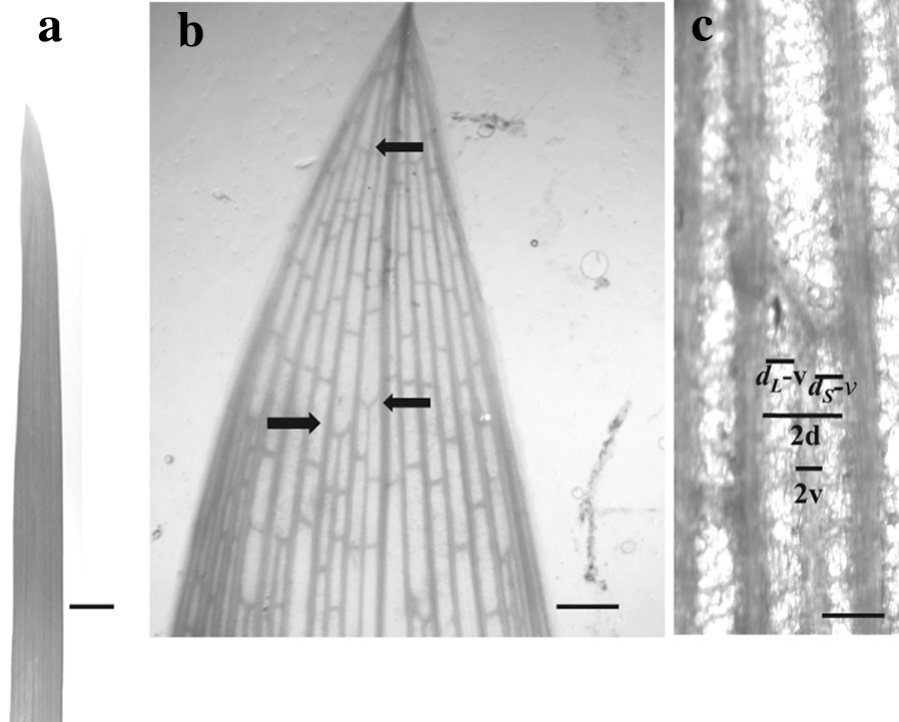
Bracts. **a** Overall shape of bract. *Bar* is 18 mm. **b** Parallel veins with occasional cross veins. Initiation of new veins (*arrows*). *Bar* is 2.8 mm. **c** Initiation of a new vein with old intervein width (2*d*), new large intervein region (*d*_*L*_-v), new small intervein region (*d*_*S*_-*v*) and new vein width (2*v*). *Bar* is 100 µm

Each plant of *Cyperus involucratus* Rottb. is composed of tough, cord-like roots bearing five to fifteen culms at any one time, some of which have flowers. Each culm bears (a) three basal scales surrounding the stem and usually free of chloroplasts, (b) a photosynthetic scape or major internode, triangular in cross section and up to two meters in length when grown in the glasshouse, and (c) 15 to 21 elongated photosynthetic bracts (Fig. 1a, b), or leaves, arranged in a trimerous phyllotaxy.

### Leaf

Longitudinal veins of both scales and bracts run parallel or are striate as they are longitudinal threads (Nelson and Dengler 1997) and at the distal end are often connected by short transverse or commissural veins (Fig. 1a–c). Typically a culm has about seven nodes with three bracts each for totals of 21 bracts each with about 20 longitudinal veins.

The degree of order of one-dimensional vein spacing as determined by the R index of Clark and Evans (1954) is 2 × 202.6 µm × (37 veins/72250 µm)^1/d=1^, or 2.06, that is, a very high degree of order. The pdf for intervein width is single-peaked with an average of 162 µm at the tip and 401 µm in the middle of the leaf (Fig. 3a). As leaf regions expand new veins are inserted between old ones bringing the intervein distance (2d) down to about half its size (d) which then continues to increase in width (Fig. 1c). Based on 25 measurements from the same bract the value of 2*d* was 228.2 µm, the larger new intervein distance averaged 114.3 µm, the smaller one of the two had a mean of 85.9 µm and the new vein was 28 µm in diameter.

### Long internode or scape

**Fig. 2:**
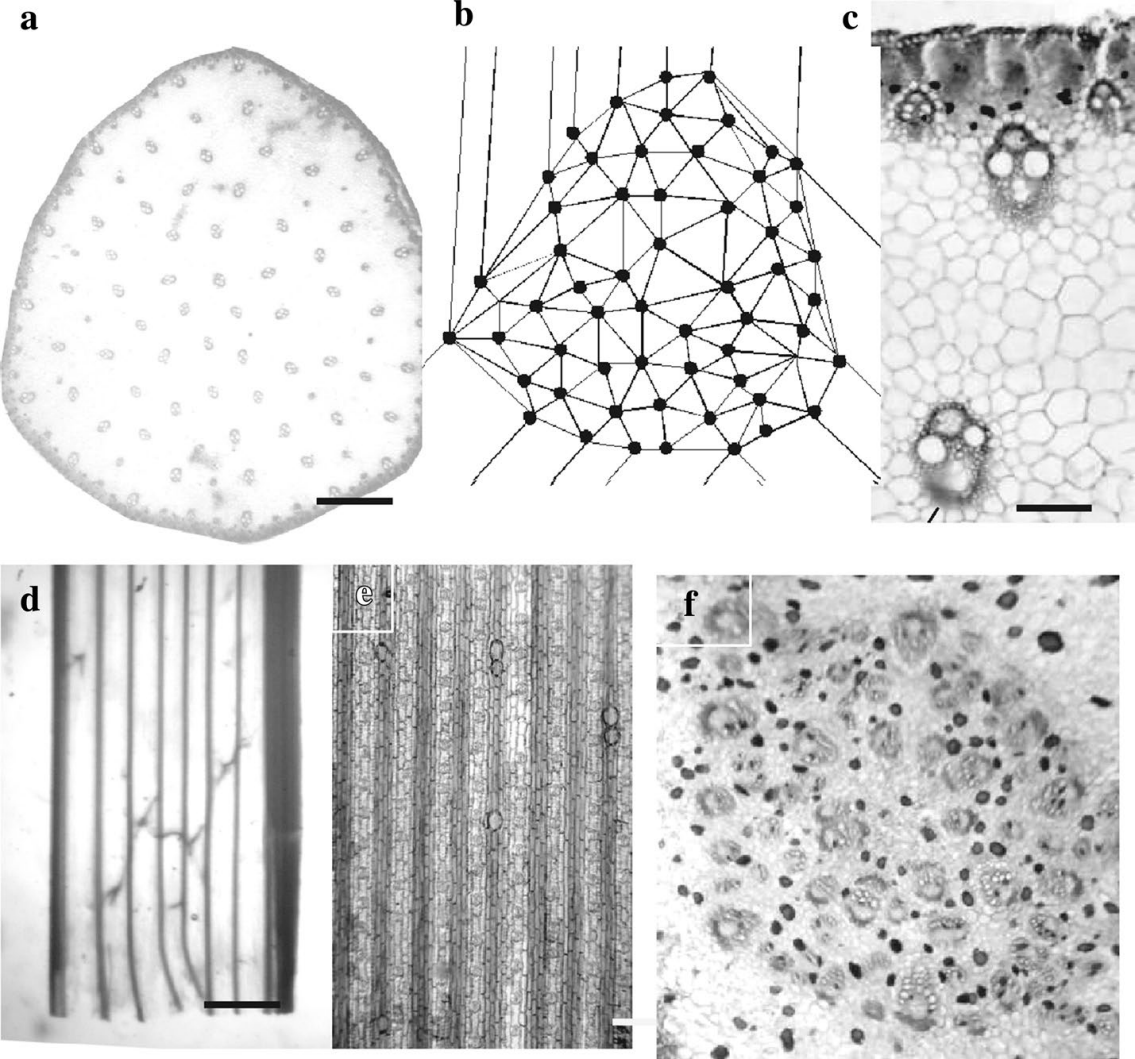
Longitudinal internode (scape) of culm. **a** Transverse view with medullary and peripheral veins. *Bar* is 1.0 mm. **b** Delaunay triangulation of vein centers with no intersecting radii. Number of radii around each center is by definition the number of nearest neighbors. **c** Two small and one large peripheral veins, one large medullary vein and three sclerenchyma bundles (above the peripheral vein). *Bar* is 150 µm. **d** Medullary veins with cross, commissural veins. *Bar* is 380 mm. **e** Peripheral veins with no cross veins. *Bar* is 300 µm. **f** Medullary veins at 7th bract internode. Veins are internally and externally (R = 0.95, random) disorganized. *Bar* is 170 µm

Green bracts are elevated by a long internode or scape often reaching two meters in height under glasshouse conditions. Scape veins are either large and well separated in the medullary, or central, region or often smaller and close together in the peripheral hypodermal chlorenchyma (Fig. 2a–c). One representative scape had 63 medullary vascular bundles for a 2-D R index of 1.67, clearly ordered and not “scattered” while the pdf for intervein distance was also single-peaked with a shoulder to the right (Fig. 4a).

This selected scape also had 109 peripheral bundles with a 1-D R index of 1.57, also clearly ordered and seemingly uniformly spaced. The pdf for peripheral bundles is single-peaked and slightly extended to the right also with a slight shoulder (Fig. 4b). Peripheral sclerenchyma strands (Fig. 2c) in the selected scape totaled 233, were strongly ordered with a 1-D R index of 1.96 and its pdf averaged 172 µm, similar to that of peripheral veins (not shown). Medullary veins extend into nodal regions and are often connected by commissural veins (Fig. 2d) while no such connections occur between peripheral veins and extend only up to the first bracts (Fig. 2e). This difference indicates medullary veins serve distal flowers and the photosynthetic leaves while peripheral veins serve the proximal photosynthetic hypodermal region of the scape.

**Fig. 3:**
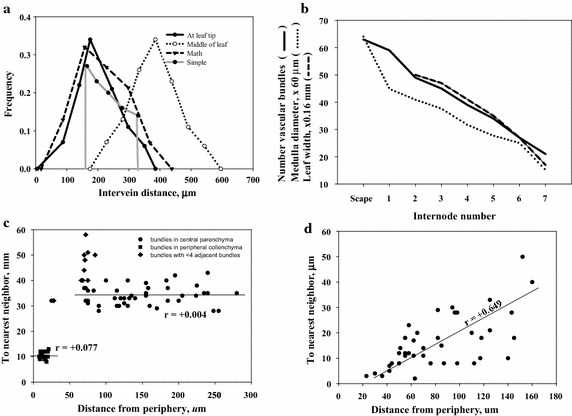
**a** Pdf data on bract intervein distances at *middle* and at tip along with data derived from the appropriate equation (Math) including binomial modification of the equation. Simple equation data. **b** Number of medullary veins, medullar diameter and leaf width at various internodes. **c** Nearest neighbor distances for medullary bundles with more than four adjacent bundles, with only four adjacent bundles and peripheral bundles in cypress. **d** Nearest neighbor distances between peripheral bundles in corn

Intervein distances in medullary and peripheral bundles seemed at times to be two patterns and at other times they appear to be one gradient pattern. Plotting intervein distances for medullary veins with at least four adjacent veins as determined by Delaunay trianglation against distance from stem periphery gave a correlation coefficient (*r*) of 0.004, indicating no correlation (Fig. 3c). Similar measurements on two stems of *Zea mays* gave an R index of 1.30, clearly but not strongly ordered, while the *r* value was 0.649, decidedly a gradient present (Fig. 3d). Based on these two cases it appears that the pattern of vein arrangement is ordered but varies from a uniform to a gradient dispersal.

Two other stem features were examined for an understanding of vein fate. First, extensive anastomosis occurs at nodes or leaf discs where leaf veins fuse with medullary veins. Also, peripheral veins and sclerenchyma bundles are absent at nodal and internodal regions indicating that they do not extend from scape to nodes and internodes.

**Fig. 4:**
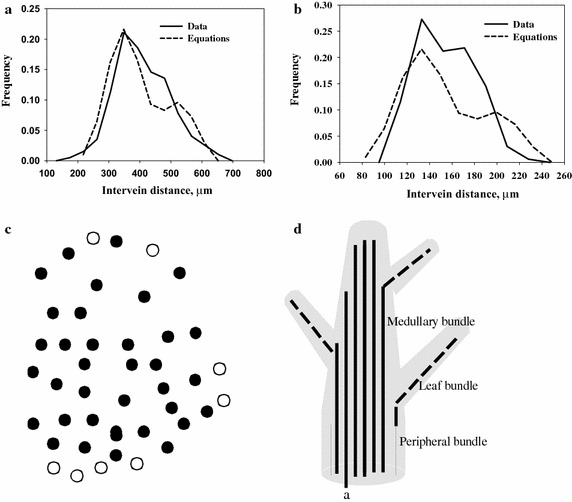
**a** Pdf for medullary stem intervein distances. **b** Pdf for intervein peripheral bundles. **c** Medullary veins of scape internode with those that extend into fifth bract internode (*filled circle*) and those that don’t (*unfilled circle*) which are located at the margin. **d** Diagram of fates of various veins with vein *a* shifting to a marginal site because of extinction of some marginal medullary veins

Second, in passing up the scape from the internode to the last formed leaves the diameter of the medullary region, the number of medullary vascular bundles and the width of leaves decreases in a linear fashion (Fig. 3b). For example, in one selected culm in passing from the scape through six bract internodes had 68, 59, 47, 36, 32, 27 and 18 medullary vascular bundles. Veins at the last internode are no longer ordered as the intervein R index is 0.95, essentially random (Fig. 2f). In particular, the loss of bundles occurs mainly at the margin of the medullary region (Fig. 4c) suggesting a type of determinate growth of the culm.

Monocot vascular patterns in leaves as well as in the medullary and peripheral stem regions are highly ordered as observed in cross section. By contrast, Zimmermann and Tomlinson (1972) found order occurs in the predictable fate of veins up the culm and into leaves, a different feature than vein relationship at any one level as studied here and that noted by others as “scattered.” The type of order identified here by the R index of Clark and Evans (1954) is from the distance between bundles at any level and so is directly related to the atactostele concept, in fact it falsifies this concept of bundles being “scattered.” In leaves the parallel arrangement seems to follow an ordered *d* to 2*d* range of intervein distances with no small distances of about vein diameter/3 thus explaining the high 1-D R index of 2.06. In stems the common description of a “scattered” arrangement of medullary veins in transverse view by Berg (1997), Purves et al., (2003) and Lima and Menezes (2009) implies no recognizable pattern is present. However, the 2-D R index of 1.67 indicates medullary veins are not randomly deployed, namely “scattered”, but are also highly ordered. Ordered one-dimensional leaf vein arrangement also holds for circular stem peripheral veins and sclerenchyma strands although the patterns are different.

The term atactostele (without order; Berbner 1902) is no longer appropriate in view of the findings that veins in stems are ordered, first, as to constant distance between adjacent veins along their length, that is, they are parallel, and, second, the intervein distances falls within a narrow range. Zimmermann and Tomlinson (1972) see the term atactostele as a “cloak of ignorance”. Presently the term eustele (true stele) is generic for eudicots and atactostele is the monocot pattern. This is a clumsy arrangement as all steles are true steles and the atactostele in Cypress stems is actually not one but two tactosteles (ordered), 1- and 2-D patterns. For eudicots the stele is a ring of vascular bundles, a 1-D configuration as is the peripheral bundle arrangement in *C. involucratus*, and taking data from Figure 11.4 of Mauseth (1988) of the buttercup stem gives a 1-D R index of 2 × 9.6 mm × 18/(53.5π))^1/1^ or 2.05, a highly ordered pattern. For the sake of clarity both monocot and eudicot patterns are considered as ordered, they are tactosteles. Given the tactostele (ordered) status for both monocot and eudicot types new terms suggested are placostele (ordered 2-dimensional, plate-like) for the former and cyclostele (ordered, one-dimensional, ring of veins) for the latter.

Two approaches were employed in taking measurements of intervein distances. First, the nearest neighbor distance used in the Clark and Evans R index is between adjacent points or centers of veins. Initially the cross-sectional developmental distance is between neighboring preprocambial cells and the preprocambial cell location becomes the center of the subsequent vascular bundle segment. The second type of measurement as in leaves also has developmental importance as it is made from the edge of one vein to that of an adjacent vein because calculations are made of the available size within which new veins might arise.

As proposed in the “Background” section the geometric description of parallel veins and the pdf of intervein distances in leaves can be extended to a more precise, mathematical expression. Intervein distance increases during leaf growth until some critical value is reached and a new vein is inserted between two adjacent veins (Fig. 1b, arrows, C). This relationship can be stated as 2*d* → *dv* + *dv* + 2*v* where 2*d* is the maximum distance between two veins (measured from edge to edge, not centre to centre), *dv* is the distance between a new and pre-existing vein and 2*v* is the width of a new vein. Based on 25 measurements from the same bract 2*d* was 228.2 µm, the larger *dv* averaged 114.3 µm, the smaller *dv* of the two had a mean of 85.9 µm and 2v was 28 µm. Intervein distance ranges from *d*v to 2*d* and increases exponentially over time *t*, or

$$ {\text{d}}_{\text{t}} = {\text{ d}}_{0} {\text{a}}^{\text{t}} \;\;\;\;0 \, \le {\text{ t }} \le { 1}0^{{}} $$

where *a* is growth rate plus 1.0 and *d*_*0*_, or *dv,* is the smallest distance. For example, if the smallest distance is 10.0 arbitrary units which increases over ten intervals of time at a rate of 0.07177 per interval then the initial distance becomes twice the original value or 20.0 units. The frequencies of these distances, f(*d*), also exponential, are

$$ {\text{f}}\left( {{\text{d}}_{\text{t}} } \right) = {\text{ d}}_{0} {\text{h}}^{t} . \, \;\;0 \, \le {\text{ t }} \le { 1}0 $$

where *h* is the decay rate of 0.9330 (the reciprocal of *a* above) and *d*_0_ is the initial frequency of 0.1145 at *t* = 0 and becomes 0.05725 when t = 10.0. Since both *d* and *f*(*d*) are dependent on *t* and are data sets, these two can be plotted with *d* on the x-axis and f(d) on the y-axis to form a doubly-truncated negative exponential distribution (Fig. 3a, simple). This f(2d)/d:f(d)/2d ratio will be referred to as the ski jump distribution.

When a new intercalary vein forms it has some width, 2*v*, so the critical distance 2*d* becomes two intervein distances and a new vein width (Fig. 4c). A probabilistic approach is considered by replacing the value of each distance d_t_ with a new distance *d* derived from a small binomial distribution $$ \left( {\begin{array}{*{20}c} 2 \\ c \\ \end{array} } \right)p^{c} q^{2 - c} $$ so d’s range from d_t_ − 1 to d_t_ + 1 or

1 $$ f\left( {d_{t} { \cdot }m} \right) = f\left( {d_{t} } \right) + \left( {\begin{array}{*{20}c} 2 \\ c \\ \end{array} } \right)p^{c} q^{2 - c} { \cdot }f\left( {d_{t + c - 1} } \right) \;\;\;\;\;t = 0  \ldots 10. $$

The resultant probability distribution from (1) of leaf intervein distances is similar to observed data when multiplying d_t_ values by *m* (Fig. 3a math).

Interestingly, the kinetics of leaf vein placement follows closely to that for size of proliferating cells (Korn 2001). A cell grows until it is *2s* in size at which time it divides into two small daughter cells of size *s* that then continue growing. The new vein in vascular kinetics replaces the new cell plate in proliferation kinetics which is negligible in thickness so *v* is dropped. Daughter cells are not equal in size but are approximately the same so initial daughter cell size is expressed by a binomial distribution.

Stem veins can also be given a mathematical description (Fig. 4a, data; b data). The orderliness of peripheral veins, that is, 1-D R is 1.57, indicating a well-spaced deployment and the calculation of pdf for distances is similar in some ways to leaf development and is different in other ways. Peripheral veins surround the internode and hence are a linear-sequenced circle so their pattern is similar to that in leaves. The difference is that the pdf is not a ski jump but is more like a children’s slide in that the frequency at 2*d* is near zero, not about half that at *d*. This difference suggests intervein distances are better measured in cell number. A simple cell pattern includes three developmental types of cells for measuring intervein distance in cell number, a preprocambial cell (*P*), a spacer cell (*S*) and a free, unspecified cell (*F*). A free cell spontaneously becomes a preprocambial cell with its adjacent cells becoming spacer cells if they were not made by earlier formed P cells. For example, a *PSFSP* sequence becomes *PSPSP* with two cases of intervein distances (*S*) being one-celled. Similarly a set of three *F* cells in a row, *PSFFFSP* becomes either *PSPSPSP* or *PSSPSSP,* namely three one-celled separation and two two-celled separations. The frequencies of one- and two-celled separations for rows of one to 10 free cell sequences as calculated empirically from a simple Monte Carlo computer model gives an oscillatory behavior of one-celled frequencies that converge to about 0.685 (Table 1, column B). Values calculated by a theoretical probability approach also fluctuate and at about 0.680 (Table 1, columns C and D).

2 $$ f\left( {d_{2 - celled} } \right) \approx 0.685\;\;\;{\text{Small distance}} $$

and the frequency of two-celled spacing*, SS*, is then 1-f (one-celled spacing), or

3 $$ f\left( {d_{3 - celled} } \right) \approx 1 - 0.685\;\;\;\;{\text{Long distance}} $$

or 0.315. While these two values from Eq. (2) are taken from the Monte Carlo method they are only approximations. For the theoretical method the question arises as to whether values should be calculated from the number of combinations or permutations. Doing the numbers for both in a three free-celled space gives values of 0.744 for the Monte Carlo method while those are 0.75 for the permutation and 0.600 for the combination methods (Table 1 row 3, columns C and D), obviously the former one of permutations is more correct. Both methods reveal an oscillatory sequence which is explained from the details of the theoretical analysis. Some odd-numbered free-celled lengths produce many permutations of all one-celled spacings whereas even-numbered spaces generates many 2-celled spacing, both of which dampen with an increase in initial free-celled lengths.

**Table 1:** Frequency of 2-celled separator space between adjacent procambial strands as calculated by (a) a Monte Carlo empirical method and (b) theoretical probability

A	B	C	D
Number free cells In a row	Empirical method	Theoretical probability	Permutations 2-celled/total
1	1.000	1.000	2/2
2	0.500	0.500	2/4
3	0.744	0.750 (0.600^a^)	6/8 (3/5^a^)
4	0.666	0.666	12/18
5	0.727	0.714	30/42
6	0.656	0.714	90/126
7	0.717	0.692	216/312
8	0.698	0708	510/720
9	0.718	0.718	1656/2304
10	0.687	0.683	4512/6600
1000	0.685	–	–

^a^If combinations not permutations are considered

Measuring distances in cell number can be converted into absolute values accordingly. First, the distance between two preprocambial cells is either one or two spacer cells but distance is from center of a preprocambial cell to that of an adjacent preprocambial cell. Since cell center to cell edge is one half of a cell and there are two of them then a one-celled distance becomes 1.0 + ½ + ½, or 2.0 cells and the two-celled distance is 2.0 + ½ + ½ or 3.0 cells. Now the one-celled distance becomes a two-celled distance and a two-celled distance becomes a three-celled distance. Distances are then two, two-celled (Small distance) and three-celled (Long distance) that together have a frequency of 1.000 or is the binomial expression of (0.69S + 0.31L)^1^ or

4 $$ {\text{f}}\left( {{\text{d}} { \cdot } {\text{m}}} \right) = \left( {\begin{array}{*{20}c} {k = 1} \\ c \\ \end{array} } \right) 0.69^{k - c} 0.31^{c} \;\;\;\;{\text{d}}\;{ = }\;{\text{c}} + {\text{S}} $$

Each cell can be either large, medium or small in size, another binomial expression, so k is increased. The pdf is single-peaked and extended to the right and, as noted earlier, is shaped like a children’s slide. The pdf for peripheral intervein distances has a k value of 5.0, the number of classes minus 1.0 of data, and an *m* value of 70 µm, to give the same mean as the data. It is clear that observed and expected data are different than that for leaf veins where the ski jump d/2f(d)-2*d*/*f*(*d*) relation holds whereas in the cell–cell scheme the *f*(2*d*) value is far less than half that at distance *d*. These are two basic types of geometric patterning, the ski jump extends over large absolute distances and the children’s slide over shorter cell–cell distances.

Beside 1-D deployment of peripheral veins and sclerenchyma bundles that for 2-D medullary veins also seems to be the cell–cell association pattern based on the low f(*d*) value at the 2*d* distance (Fig. 4a). Generally, nearest neighbor distances in 2-D space are linear measurements so linear measurement of cell number between adjacent veins seems justified. Hence arrangement of trichomes, cotton fibers and some cases of stomata seem to be examples of cell–cell interaction as they appear to be separated by either one or two cells at maturity. Thus Eqs. (2) and (3) describe the 2-D pattern of these epidermal derivatives and by Eq. (4) for peripheral and sclerenchyma bundles.

The loss of marginal vascular bundles in the medullary region over consecutive internodes has some consequence on the fate of the more central medullary bundles. Zimmermann and Tomlinson (1972) found vascular bundles viewed up the culm often branch into leaves while shifting from central to more marginal positions. The shift here in *Cyperus* occurs because marginal vascular bundles are lost leaving central bundles to assume more marginal positions (Fig. 4d).

All three types of description, geometric, data and mathematical, avoid inferring what the molecular mechanism of patternization might be. A good description should not be biased in impling any mechanism. These ordered, well-spaced patterns might be the expression of the canalization system of Sachs (1981) or the diffusion reaction model of Glierer and Meinhardt (1972) but neither has been applied to any specific histological data. The theory of simple diffusion of an inhibitor (Veen and Lindenmayer 1977) has been applied only to phyllotaxy successfully while the coordinated growth concept (Korn 1993) or the cell-to-cell induction hypothesis (Korn 2008) explains some aspects of vein formation. These five hypotheses need to be extended to quantitative descriptions for predicting specific features of some geometric trait such as distance between parallel veins. Recently, Carteri et al. (2014) developed a diffusion–reaction model that generates the geometries of most stellar patterns, i.e., eustele (now tactostele) and protostele but not the atactostele (now placostele). Also, their model mimicks only general geometries but not stelar data. It is therefore essential that data from tissue and from a model is collected on parameters useful for testing. Until then, the simplest ones, that of cell-to-cell induction and inhibitor diffusion, require only spacing by diffusion of an inhibitor in one-, two- or three-D’s, so are tentitively adapted here for the case of monocot parallel venation.

Mutant leaves offer an opportunity to test the various hypotheses of vein patternization. The *midribless* mutants reported in barley (Seip and Tauchiya 1979), pearl millet (Appa Rao et al. 1989), *Panicum* (Fladung 1994) and maize (Landoni et al. 2000) are interesting but are without a keel that is typically located beneath an average-sized normal vascular bundle. Far more interesting for testing hypotheses is displacement of leaf veins which may be farther apart (Fladung 1994) or closer together (Landoni et al. 2000). Scarpella et al. (2003) found the *RAL1* mutation in rice leads to numerous changes including smaller distances between longitudinal leaf veins. Data on intervein distances for all three cases are wanting. Vein displacement alteration can easily be incorporated into Eq. (4) by a simple change in the value of *d*.

Venation in *C. involucratus* stem and leaf involves two separate patterns. One is the separation of bundles over large absolute distances (or many-celled), expressing the ski jump pdf, and the other is close, cell contact arrangement generating the children’s slide pdf. Also each can be defined mathematically by equations so descriptions pass beyond the crude geometric stage. It is of interest to examine other monocots for vascular pattern, whether it has a uniform distribution (Fig. 3c) or a gradient profile (Fig. 3d) or possibly some other distribution. A more deeply seated phenomenon is the internal organization of vascular bundles with phloem facing the outside in stems or the adaxial surface of leaves and xylem located to the center of stems and the abaxial face in leaves. Several observed exceptions in stems to this rule suggest a relationship between internal (within a bundle) and external (between bundles and stem margin) with the latter serving as an *entre* to the problem of the former.

## Conclusions

The data collected here clearly indicates the 2D arrangement of vascular bundles in the medullary region of the scape of *Cyperus* is highly ordered in contrast to the “scattered” description noted by many others. The linear deployments of stem peripheral vascular bundles and sclerenchymatous bundles along with leaf vascular bundles are also clearly ordered.

Patterns of these four bundle arrangements are extended from simple, crude geometries, i.e., parallel, evenly spaced, etc., to a mathematical descriptions void of causal assumptions. The equation for the leaf pattern is similar to that for sizes of proliferating cells and that for medullary veins can also be applied to those for epidermal derivatives such as stomata, hairs and cotton fibers.

The term atactostele, meaning without order, is no longer appropriate and new terms are suggested, placostele for monocot stems and cyclostele for eudicaot stems.

## Methods

Seven clonal plants of *Cyperus involucratus* Rottb. having about 105 culms in various stages of development were growing in the university glasshouse. Material was made as either permanent sides fixed in FAA, run through an alcohol-xylene-paraffin series, sectioned at 12 µm and stained with safranin and fast green or temporarily prepared as hand-sections at about 100 µm and treated with phloroglucinol and then concentrated HCl.

The degree of order of vascular patterns was measured by the R index of Clark and Evans (1954) by the expression $2n\sqrt[d]{D}$ where *n* is the average nearest neighbor distance, *d* is the number of dimensions of the pattern and *D* is the density which is the number of loci in a specified area. A value of 2.14 is perfect ordering (hexagonal bathroom tiles), 1.0 is random (large raindrops on a sidewalk) and 0 is clustering (ballroom dance partners). Veins were observed from cross sections and adjacent veins were identified by Delaunay triangulation using a computer program from the internet (Voronoi Diagram/Delaunay Triangulation Applet). Sample size of data is 400 unless specified.

For sake of comparison the stem vascular bundle arrangement was also examined in two permanent slides of *Zea mays* (George Conant, Ripon WI. and Carolina Science, Burlington, NC, USA).
